# Out of Physic: Into—What?

**Published:** 1890-03-29

**Authors:** 


### THE DOCTOR'S PULPIT.

### OUT OF PHYSIC: INTO—WHAT?

IN mediæval times it was a not uncommon thing for the monks to discuss, over the walnuts and the wine, the very recondite question—"How many angels could stand upon the point of a needle?" That problem, like a great many others that engaged the attention of the philosophic minds of the middle ages, still remains unsolved. We do not now propound it as a topic for the consideration of Medical and Clinical Societies, either metropolitan or provincial; though as a variation on the eternal "Microbe" question it might be not without its attractions. Probably nobody will ever know how many angels can stand on the point of a needle; but we can find out with the nicest exactitude, by one of the methods of "original research," how many middle-aged doctors, with their wives and families, can stand on a wooden platform of a given length and width. The platform, being of wood and inelastic, will hold a certain number with ease; it will hold a few more by a little judicious arranging of the separate individuals; but there comes a time when every available inch of space is occupied; and when that period is reached the attempt to add more will cause some either to jump off voluntarily, or to tumble, *nolens volens*, into surrounding space.

The platform to which we have likened the standing ground of the medical profession has long been too full for the comfort of its occupants, and at the present time it is so packed, and so many new claimants are demanding a foothold upon it, that we feel we cannot render a better service to our medical confrères than by pointing out to them how some of the more adventurous may safely and wisely take a leap and land themselves on more comfortable and profitable standing ground.

A patient will often remonstrate with his doctor when the latter advises a day or two in bed or a few weeks' rest and change. "My business will not allow me," says he, "to] be absent; I must be at my office whether I am well or ill." To which the man of physic replies, in tones Johnsonian, "Sir, illness takes no account of business necessities; if a man have typhoid fever or diphtheria he must stay at home and in bed, though bankruptcy should stare him in the face in consequence." But if pathological science takes no account of a patient's business necessities, neither does the science of political economy, represented in this case by the twin monsters of supply and demand, take any account of the doctor's family difficulties. What do supply and demand care though the family physician be a man of excellent morals and of untiring industry; though he begin his work early in the morning and leave it late at night; though he rise at the ringing of the midnight bell and return to his rest with the grey dawn; though he cut down his fees to a minimum, and lose a fourth or even a third of his earnings in bad debts; though he wear old hats and turned coats, and dress his wife and children in the cheapest of new garments and in the most faded and dyed of old ones; though he live in a low-rented house whose appearance is too mean for dignity and whose rooms are too small for health; though his children's education is more deficient than his own, and their start in life lower down the scale; though he is likely to be poorer at sixty than he was at thirty, and at seventy to be partly or wholly dependent upon others—What, we say, does the law of supply and demand care for all these things? Nothing! Absolutely nothing at all.

Emigration, exodus, is the panacea of statesmen and political economists for overcrowded countries. It is a good remedy, but it needs a certain amount of courage and will on the part of the emigrant. How many doctors are there who would march out of their professional positions to-morrow if they could be certain, or even reasonably hopeful, of marching into a modest sufficiency of bread and butter, with plain clothes, and a less harassing occupation! Not many years ago it was generally felt that there must be either an exodus from the existing ranks of medicine or a barrier raised against the inflow of more doctors. The feeling now is that even though there were to be a check to the inflow there would still need, to make things tolerable, a large exodus in addition. The body medical must be depleted, and that by drastic measures, or the consequences will be fatal to a good many of its members.

Englishmen nowadays do not seem to have either the courage or the mental versatility of their Anglo-Saxon, Danish, and Norman forefathers. Those ancient heroes, finding life too straitened for them on the continent, boldly swooped down on this country and made new homes for themselves by the right of the survival of the fittest. The modern man says he cannot carve out a new career by means of his sword. No! neither would they, if they could have found out any safer or less cut-throat way. The question for the modern man of education is not whether he can make a new career for himself by means of the sword, but whether there is any possibility of his making a new career at all, by any means. Granting that doctors are too thick on the ground for the plainest of living and even for health, is there any opening for the younger and more adventurous of them, either in this or any other country, which gives promise of more health, more happiness, and more success than the practice of medicine in Great Britain? Before answering this question another may be asked: What is the young doctor fit for? By "young" we mean under forty years of age. Let us go back a little on his past history. Medical students are drawn largely from villages and small towns. They are the sons of clergymen, doctors, the better class of farmers, and sometimes of successful shopkeepers. They are familiar with the country and country pursuits. They know about corn growing, horse breeding, cattle rearing, fruit cultivation, and all kinds of gardening. As medical students they study not only human anatomy, but comparative; they become familiar with the structure, life history, and habits of the lower animals. They learn something of elementary chemistry, too. In short they deal, at any rate in books, with soils, climates, and every variety of animal life. Have they any sympathy with, or real understanding of the facts of natural science with which they are thus brought into intelligent contact? If they have, it is plain that whatever else they are not fit for they are at least in some sense fit for dealing with land and live stock. No doubt many of them will say here that though they have some book knowledge they have no practical experience. But how much practical experience has the newly qualified doctor of the treatment of patients, which he undertakes with such a light heart? The want of experience does not prevent him making experiments upon other people's health and life, and charging fees for the same. Why then should he not put his scientific knowledge to the test on soils and animals, and at the expense of his own capital?

The objector thinks he can close the argument in his own favour by asking two questions. Where is the land he is to take to? And how much profit is he to make for his labour and capital? With regard to the land there is no need nowadays to cross the seas in search of it. It lies at the very doors of every man in every county in England; and it can be had for a merely nominal sum either to rent or to purchase. The second question demands a little more space in the answering. When the objector asks how much profit is to be made out of land, we must ask him what he means by "profit." Will any educated man regard money as the only conceivable "profit"? Is health not profit? Is sound sleep not profit? Is power of work up to old age not profit? Is a wife whose face is unwrinkled with care not profit? Is a robust and merry family of children not profit? It is astonishing, beyond all power of words to express, how narrow-minded, conventional, cowardly, and destitute of originality educated men can become even in the course of a dozen years. But admitting, for the sake of the argument, that "money" represents to the average man the principal part of the "profit" to be looked for in life, we maintain that the young doctor who could farm scientifically would in these times make more actual profit out of land than he would as a general medical practitioner, if he devoted the same untiring energy and the same initial capital to the business as the ordinary general practitioner is compelled to devote to his practice.

